# Short-term outcomes of robotic vs. laparoscopic surgery for rectal cancer after neoadjuvant therapy: a meta-analysis

**DOI:** 10.3389/fsurg.2023.1292031

**Published:** 2024-01-11

**Authors:** Yuqiang Zhang, Bo Dong, Guanglin Li, Wei Ye

**Affiliations:** Department of General Surgery, People’s Hospital of Rongchang District, Chongqing, China

**Keywords:** robotic surgery, conventional laparoscopic surgery, rectal cancer, neoadjuvant therapy, meta-analysis

## Abstract

**Background:**

The effect of robotic surgery (RS) for rectal cancer after neoadjuvant therapy is still controversial, and a comprehensive search and analysis of the current relevant evidence is necessary. Our study aimed to evaluate the efficacy of RS for rectal cancer after neoadjuvant therapy compared with conventional laparoscopic surgery (LS).

**Methods:**

Up to August 23, 2023, Embase, PubMed, Web of Science, and Cochrane databases were searched for studies of RS for rectal cancer after neoadjuvant therapy. Odds ratio (OR) or mean difference (MD) was used to calculate the effect sizes using RevMan 5.3.

**Results:**

A total of 12 studies reporting on 11,686 participants were included. Compared with LS, RS increased the operative time (MD 35.16 min; 95% CI: 16.24, 54.07), but it did significantly reduce the risk of the conversion to open surgery (OR 0.46, 95% CI 0.40, 0.53) and improved the TME incomplete rate (OR 0.40, 95% CI 0.17, 0.93). Moreover, there were no difference in total postoperative complications (OR 1.13, 95% CI 0.84, 1.52), circumferential resection margin positivity (OR 0.90, 95% CI 0.63, 1.27), distal margin positive (OR 0.60, 95% CI 0.29, 1.22), blood loss (MD −11.57 ml; 95% CI: −39.09, 15.94), length of hospital stay (MD −0.08 days; 95% CI: −1.26, 1.10), mortality (OR 0.59, 95% CI 0.29, 1.21), lymph node harvested (MD 0.69.; 95% CI: −0.43, 1.82), and the time of first flatus (MD −0.47 days; 95% CI: −1.19, 0.25) between the two groups.

**Conclusions:**

RS was associated with superiority over LS in reducing the risk of the conversion to open surgery and improving TME incomplete rate, which suggested that RS could be an effective method for treating rectal cancer after neoadjuvant therapy.

**Systematic Review Registration:**

https://www.crd.york.ac.uk/PROSPERO/display_record.php?RecordID=460084, PROSPERO (CRD42023460084).

## 1 Introduction

Colorectal cancer is the third most common cancer and one of the most common causes of cancer death worldwide. About 40% of colorectal cancers occur in the rectum (1, 2). Surgical resection is the main treatment strategy for rectal cancer. Compared with open surgery, laparoscopic minimally invasive surgery has the advantages of shorter operation time, faster postoperative recovery and fewer complications, and is becoming more and more popular among surgeons (3, 4). However, there are some limitations of the laparoscopic surgery (LS), such as anatomical limitations of the narrow pelvis, amplification of the handle tremor, and unstable images provided by the hand-held camera of the assistant (4). Preoperative chemoradiotherapy can effectively reduce the tumor stage, circumferential resection margin positive rate and local recurrence rate (5). However, neoadjuvant chemoradiotherapy can cause tissue edema and fibrosis, which will lead to blurred dissection plane and increase the difficulty of surgery (6).

Robotic surgery (RS) is expected to overcome these limitations by providing a 3D view of the environment, flexible operating instruments and a stable camera platform (4, 5). A large number of studies have explored the application of RS in rectal cancer surgery, but there are limited studies on the application of RS in patients receiving neoadjuvant therapy. In addition, several current studies provide conflicting evidence. Yamanashi et al. (7) demonstrated that RS reduced the incidence of postoperative complications and shortened the length of postoperative hospital stay in patients with neoadjuvant rectal cancer compared with LS. However, the study by Chen et al. (8) found that the incidence of postoperative complications in the robotic group was similar to that in the laparoscopic group. In addition, RS resulted in increased operative time and blood loss.

To resolve the conflicting results of current studies and overcome the lack of high-quality evidence, we conducted a comprehensive literature search and conducted a meta-analysis of currently published data to evaluate the differences in postoperative short-term outcomes between RS and LS in rectal cancer patients after neoadjuvant therapy.

## 2 Methods

### 2.1 Search strategy

A systematic search for the current meta-analysis was conducted on material published from inception, to August 23, 2023, in the Embase, Web of Science, PubMed, and Cochrane Library databases, using the following search string: (rectal cancer OR rectum cancer OR rectal tumor OR rectum tumor OR rectum neoplasm OR rectal neoplasm) AND (laparoscopic OR laparoscopy OR laparoscope) AND (robotic OR robotics OR robotic-assisted OR robot-assisted) AND (neoadjuvant OR neoadjuvant chemoradiotherapy OR neoadjuvant chemotherapy OR neoadjuvant radiotherapy) (Table 1). No language restrictions were imposed. The list of references for the relevant reviews and included studies were also searched.

**Table 1:** Electronic search strategy.

Database	Search term	Number
PubMed (All fields)	#1: rectal cancer OR rectum cancer OR rectal tumor OR rectum tumor OR rectum neoplasm OR rectal neoplasm	#1: 97,274
	#2: laparoscopic OR laparoscopy OR laparoscope	#2: 1,74,160
	#3: robotic OR robotics OR robotic-assisted OR robot-assisted	#3: 90,967
	#4: neoadjuvant OR neoadjuvant chemoradiotherapy OR neoadjuvant chemotherapy OR neoadjuvant radiotherapy	#4: 56,356
	#5: #1 AND #2 AND #3 AND #4	#5: 149
Embase (All fields)	#1: rectal cancer OR rectum cancer OR rectal tumor OR rectum tumor OR rectum neoplasm OR rectal neoplasm	#1: 1,63,110
	#2: laparoscopic OR laparoscopy OR laparoscope	#2: 2,98,876
	#3: robotic OR robotics OR robotic-assisted OR robot-assisted	#3: 1,27,170
	#4: neoadjuvant OR neoadjuvant chemoradiotherapy OR neoadjuvant chemotherapy OR neoadjuvant radiotherapy	#4: 1,03,651
	#5: #1 AND #2 AND #3 AND #4	#5: 441
Cochrane Library Trials (All fields)	#1: rectal cancer OR rectum cancer OR rectal tumor OR rectum tumor OR rectum neoplasm OR rectal neoplasm	#1: 8,967
	#2: laparoscopic OR laparoscopy OR laparoscope	#2: 26,296
	#3: robotic OR robotics OR robotic-assisted OR robot-assisted	#3: 7,173
	#4: neoadjuvant OR neoadjuvant chemoradiotherapy OR neoadjuvant chemotherapy OR neoadjuvant radiotherapy	#4: 11,799
	#5: #1 AND #2 AND #3 AND #4	#5: 19
Web of Science (All fields)	#1: rectal cancer OR rectum cancer OR rectal tumor OR rectum tumor OR rectum neoplasm OR rectal neoplasm	#1: 78,705
	#2: laparoscopic OR laparoscopy OR laparoscope	#2: 1,70,931
	#3: robotic OR robotics OR robotic-assisted OR robot-assisted	#3: 1,69,053
	#4: neoadjuvant OR neoadjuvant chemoradiotherapy OR neoadjuvant chemotherapy OR neoadjuvant radiotherapy	#4: 67,821
	#5: #1 AND #2 AND #3 AND #4	#5: 142

### 2.2 Study selection

The prespecified PICO criteria were: (1) patients: patients undergoing rectal cancer surgery after neoadjuvant therapy, (2) intervention: intervention with robotic rectal resection, (3) comparator: compare with laparoscopic rectal resection, (4) outcome: the outcomes included any of the following: postoperative complications, surgical margins, operative time, length of hospital stay, the time of first flatus and defecation, mortality, lymph node harvested, blood loss, conversion rate, and total mesorectal excision (TME) completeness, (5) study design: randomized controlled trials (RCTs) and comparative non-randomized studies (NRS). Primary outcome indicator was the incidence of postoperative complications, and the secondary outcome were postoperative complications, surgical margins, operative time, length of hospital stay, the time of first flatus and defecation, mortality, lymph node harvested, blood loss, conversion rate, and TME completeness.

Exclusions contain: review, conference abstracts, case reports, letters to the editor, technical reports, single arm studies, and non-human studies.

### 2.3 Data extraction

Data, including study type, country, sample size, age, neoadjuvant therapy, sex, the first author, year of publication, intervention type, control groups, and outcomes were extracted from each study. If relevant data could not be extracted from the literature, we tried to contact the corresponding author of the study to obtain relevant information.

### 2.4 Quality assessment

Methodological quality of RCTs was assessed using the Cochrane Collaboration's risk-of-bias tool (9): (1) the randomizing process, (2) allocation concealment, (3) participant and operator blinding, (4) blinding of outcome assessment, (5) incomplete data, (6) selective reporting, and (7) other biases. the Newcastle–Ottawa scale (NOS) was used to evaluate the quality of NRS. The score of NOS is 9 points. Scores ≥ 6 were considered high quality studies, and scores < 6 were classified as low quality studies. Two authors (Ye and Zhang) independently conducted literature retrieval, study selection, data extraction, and quality assessment of the methodology included in the study. When there were inconsistencies between the two authors, they were discussed and resolved by a third author (Ye, Dong and Zhang).

### 2.5 Statistical analysis

Odds ratio (OR) with 95% confidence intervals (CI) were calculated for qualitative variables and mean difference (MD) for quantitative outcomes. If median with range or interquartile was reported, mean and SD were calculated using the formula reported by Wan et al. (10) and Luo et al. (11). Heterogeneity between studies was assessed using the *I*^2^ statistic (12). The random effects model was selected when *I*² was >50%. Otherwise, the fixed-effect model was selected. To explore the robustness of the results, one-study exclusion test was used to examine the impact of each study on the pooled effect size. Analysis was conducted using Review 5.3 (The Nordic Cochrane Centre, The Cochrane Collaboration 2014; Copenhagen, Denmark). *P* < 0.05 was considered significant.

## 3 Results

### 3.1 Selected studies

**Figure 1:**
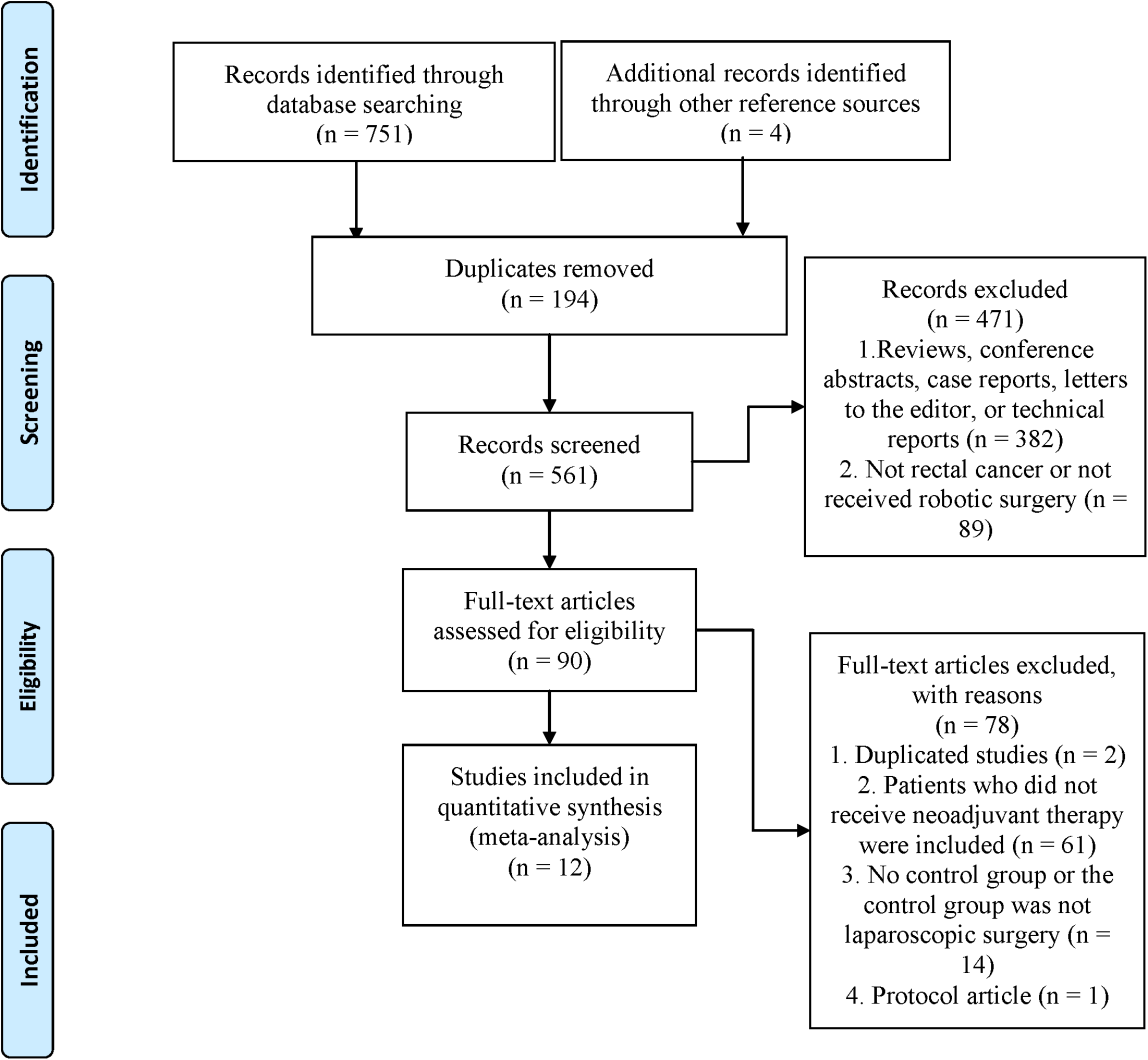
Flow chart of literature search and screening.

We initially identified 755 articles. 561 were retained after 194 duplicates were excluded. 471 articles were excluded by reading titles and abstracts, and the remaining 90 articles were evaluated for full text. Finally, twelve trials (5–8, 13–20) were eligible and included for meta-analysis (Figure 1).

### 3.2 Study characteristics and quality assessment

All the 12 included studies were NRS, and three studies (7, 8, 18) adopted propensity score matching. Twelve studies (5–8, 13–20) with a total of 11,686 participants (RS = 3,954, LS = 7,732) were published between 2013 and 2023. Three of the studies were conducted in China (6, 8, 15), three (13, 18, 20) in South Korea, two (16, 17) in the United States, two (5, 7) in Japan, and the remaining two in Turkey (14) and Switzerland (19), respectively. The quality assessment scores of the included studies are summarized in Table 2, with all studies scoring between six and nine.

**Table 2:** Characteristics of 12 eligible studies.

First author, year	Country	Study design	Sample	Age	Gender (M/ F)	Neoadjuvant therapy	Outcomes	NOS
Saklani, 2013	South Korea	Retrospective cohort study	R: 74	R: 59.6	R: 50/24	LCRT	Hospital stay, postoperative complications, mortality, conversion rate, operating time, blood loss, the time of first flatus, CRM, lymph node harvested	8
			L: 64	L: 60.1	L: 46/18			
Serin, 2015	Turkey	Retrospective cohort study	R: 14	R: 54 (41–71)	NA	LCRT/SCRT	Postoperative complications, CRM, operating time, hospital stay, lymph node harvested, conversion rate, TME completeness, the time of first flatus	7
			L: 65					
				L: 57 (28–80)				
Huang, 2017	China	Retrospective cohort study	R: 40	R: 60	R: 25/15	CRT	Postoperative complications, operating time, blood loss, hospital stay, CRM, the time of first flatus, conversion rate	7
			L: 38	L: 60.1	L: 28/18			
Sujatha-Bhaskar, 2017	USA	Retrospective cohort study	R: 905	R: 57	R: 65/35	CRT	Mortality, CRM, conversion rate, lymph node yield	7
			L: 2,009	L: 57	L: 61/39			
Hopkins, 2020	USA	Retrospective cohort study	R: 2,472	R: 59 (51–68)	R: 1,637/835	CRT	Mortality, hospital stay, lymph node yield	7
			L: 5,144		L: 3,196/1,948			
				L: 69 (51–68)				
Park, 2021	South Korea	PSM, retrospective cohort study	R: 118	R: 60.3	R: 90/28	CRT	Postoperative complications, CRM, DRM, operating time, hospital stay, conversion rate	8
			L: 118	L: 60	L: 87/31			
Angehrn, 2022	Switzerland	Retrospective cohort study	R: 38	R: 66 (57–79)	R: 29/9	CRT	Postoperative complications, mortality, CRM, TME incomplete, operating time, hospital stay, conversion rate, lymph node yield	8
			L: 64		L: 42/22			
				L: 63 (56–72)				
Chen, 2022	China	PSM, retrospective cohort study	R: 56	R: 57.4	R: 39/17	LCRT	Postoperative complications, CRM, DRM, operating time, blood loss, hospital stay, conversion rate, lymph node yield	8
			L: 56	L: 56.3	L: 38/18			
Piozzi, 2022	South Korea	Retrospective cohort study	R: 124	R: 55 (47–65)	R: 86/38	LCRT/SCRT	Postoperative complications, mortality, CRM, TME completeness, operating time, blood loss, hospital stay, conversion rate, the time of first flatus and defecation, lymph node yield	7
			L: 36		L: 28/8			
				L: 60.5 (52.5–67)				
Yamanashi, 2023	Japan	PSM, retrospective cohort study	R: 30	R: 62.4	R: 24/6	LCRT	Mortality, operating time, blood loss, hospital stay, conversion rate, lymph node yield	8
			L: 30	L: 61.7	L: 26/4			
Lim, 2023	Japan	Retrospective cohort study	R: 46	R: 61	R: 29/17	LCRT	Postoperative complications, operating time, blood loss, lymph node yield	7
			L: 64	L: 63	L: 43/21			
Zhang, 2023	China	Retrospective cohort study	R: 37	R: 58.1	R: 11/26	LCRT	Lymph node yield, postoperative complications, mortality, operating time, blood loss, the time of first flatus	8
			L: 44	L: 57.6	L: 21/23			

CRT, chemoradiotherapy; CRM, circumferential resection margin; DRM, distal resection margin; F, Female; L, laparoscopic surgery; M, male; LCRT, long-course chemoradiotherapy; NOS, newcastle-ottawa scale; PSM, propensity score matching; SCRT, short-course radiotherapy; R, robotic surgery; TME, total mesorectal excision; UK, United Kingdom; USA, United States of America.

### 3.3 Meta-analysis

#### 3.3.1 Postoperative complications

**Figure 2:**
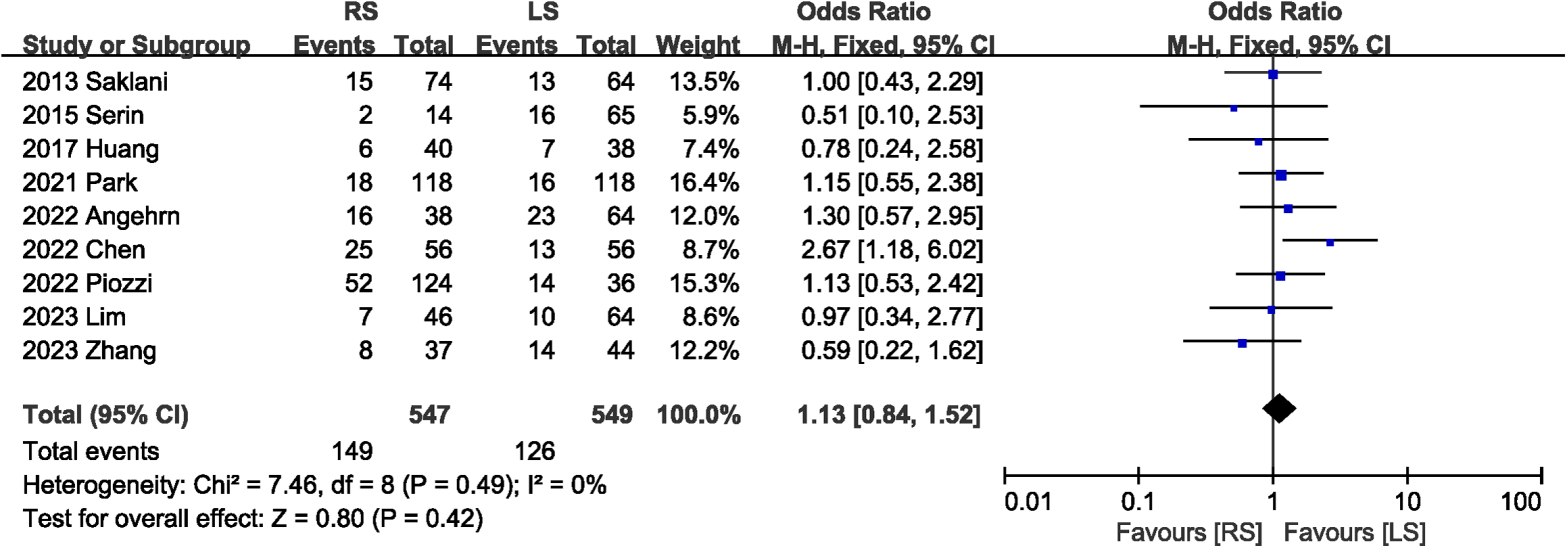
Forest plot of postoperative complications.

A total of 1,096 participants in nine studies (5, 6, 8, 13–15, 18–20) mentioned postoperative complications. The combined results suggested that the total postoperative complications were comparable between the RS and LS groups, with low heterogeneity (OR 1.13, 95% CI 0.84, 1.52; Heterogeneity: *I*^2^ = 0%, *P* = 0.49) (Figure 2).

#### 3.3.2 Conversion rate

**Figure 3:**
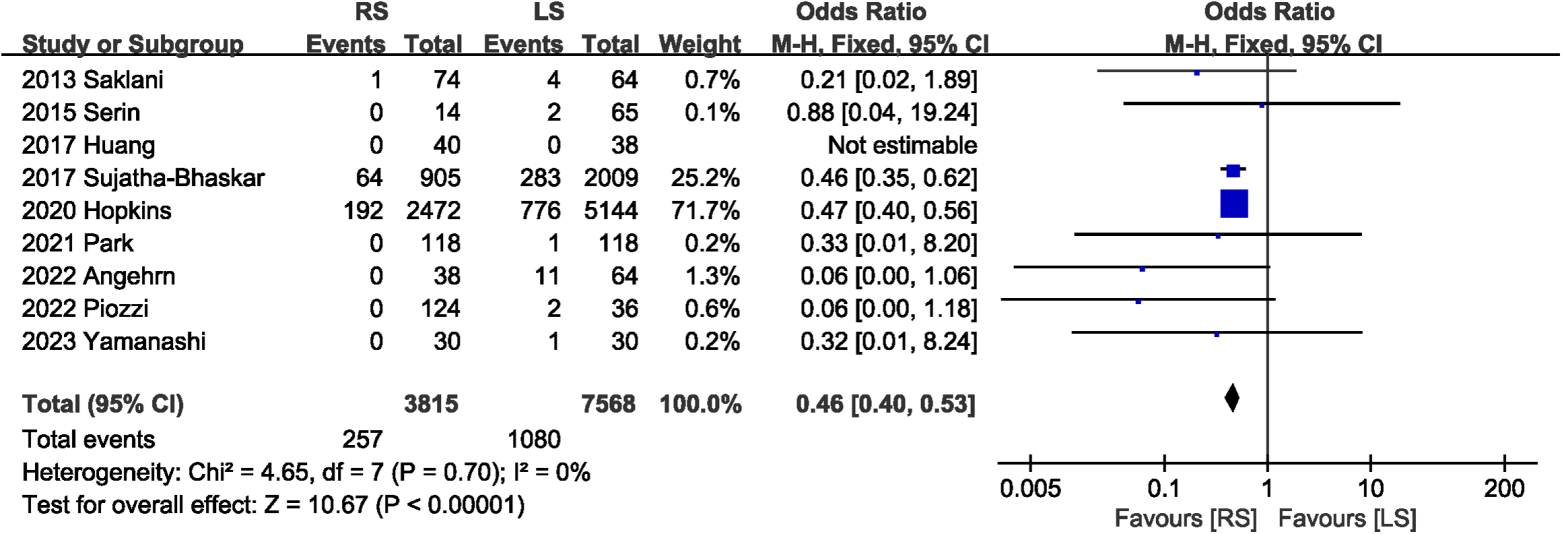
Forest plot of conversion rate.

Incidence of conversion to open surgery was reported in nine studies (7, 13–20). Compared with LS, RS was associated with a lower risk of conversion to open surgery (OR 0.46, 95% CI 0.40, 0.53) (Figure 3). No significant heterogeneity (*I*^2^ = 0%, *P* = 0.70) was observed between studies.

#### 3.3.3 Mortality

**Figure 4:**
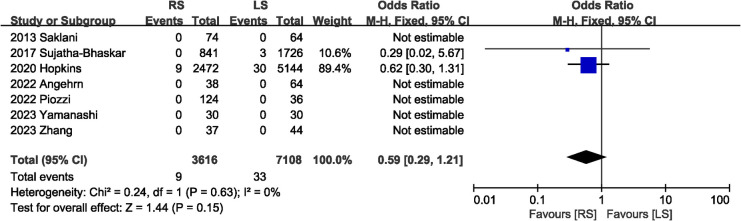
Forest plot of mortality.

Postoperation mortality data were recorded in seven studies (6, 7, 13, 16, 17, 19, 20), five of which had no deaths in the RS and LS groups. The pooled results showed no significant difference in mortality between the RS and LS groups (OR 0.59, 95% CI 0.29, 1.21; Heterogeneity: *I*^2^ = 0%, *P* = 0.63) (Figure 4).

#### 3.3.4 Operative time and intraoperative estimated blood loss

**Figure 5:**
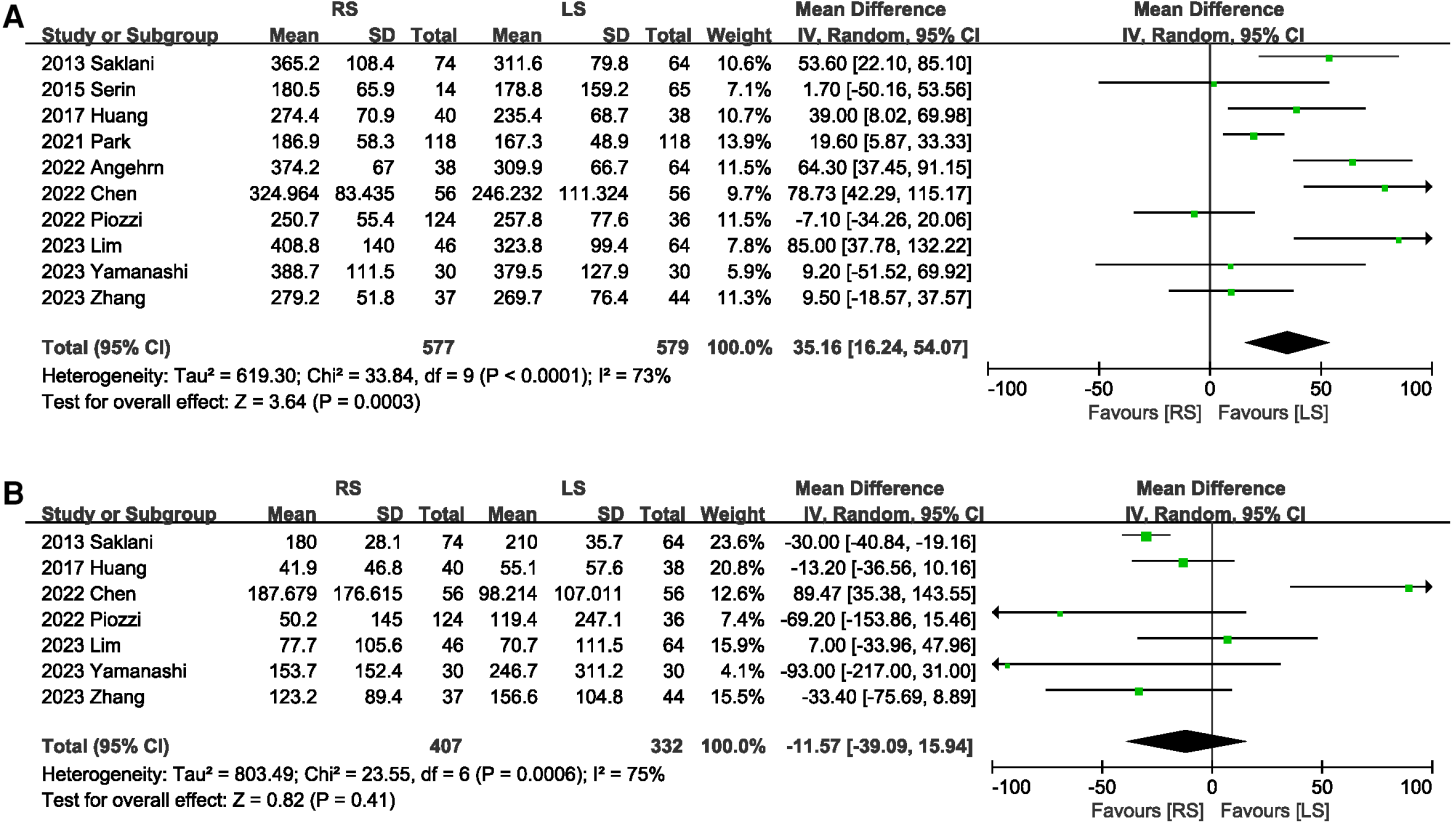
Forest plot of (**A**) operative time and (**B**) intraoperative estimated blood loss.

Ten studies (5–8, 13–15, 18–20) described the operative time. Compared with LS, RS significantly prolonged the operation time (MD 35.16 min; 95% CI: 16.24, 54.07; Heterogeneity: *I*^2^ = 73%, *P* < 0.0001) between studies (Figure 5A). The pooled data from seven studies (5–8, 13, 15, 20) showed that the blood loss in RS was similar to that in LS (MD −11.57 ml; 95% CI: −39.09, 15.94; Heterogeneity: *I*^2^ = 75%, *P* = 0.0006) (Figure 5B).

#### 3.3.5 Pathological outcomes

**Figure 6:**
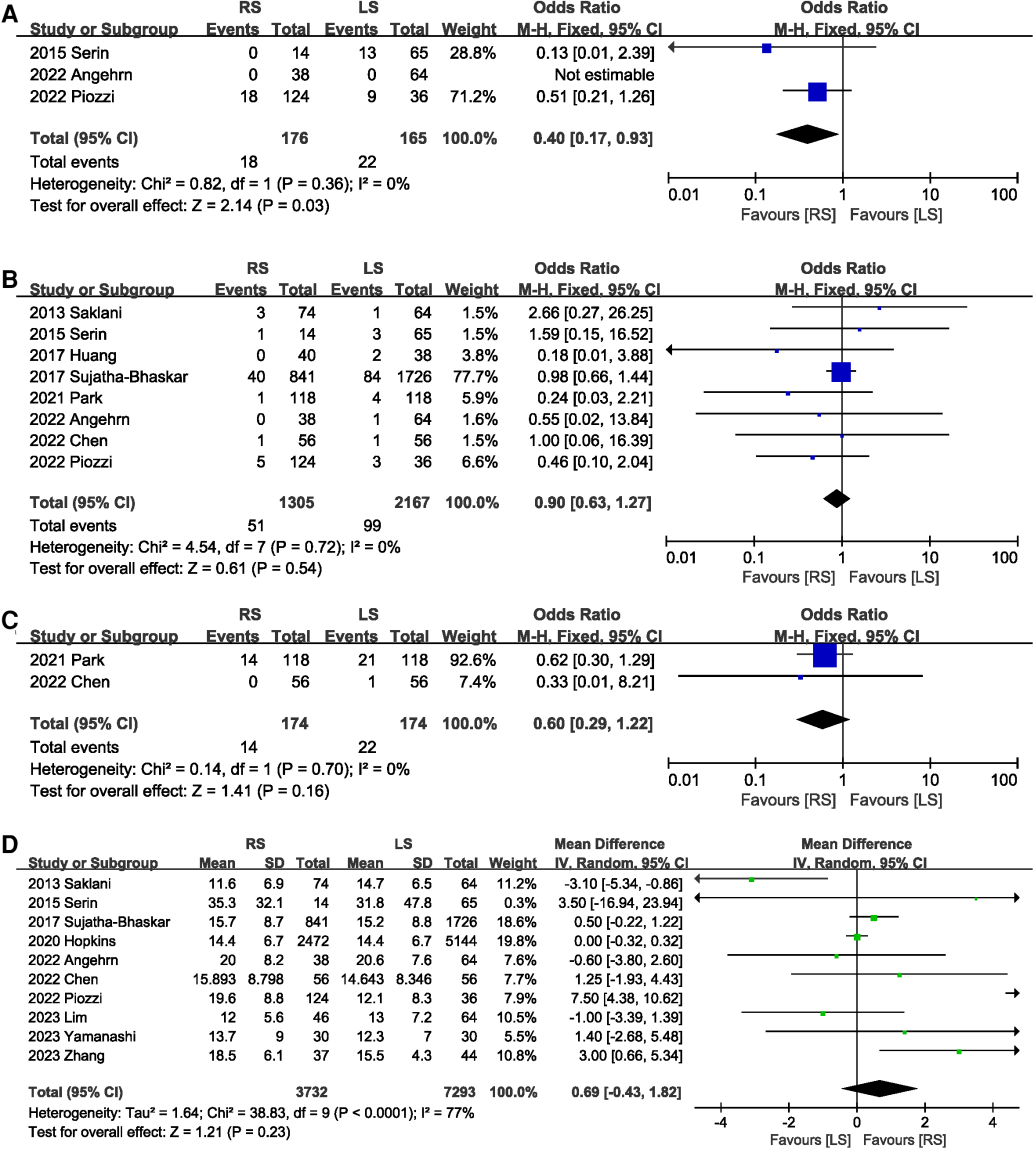
Forest plot of (**A**) TME incomplete, (**B**) CRM positivity, (**C**) distal margin positive rates, and (**D**) the number of nodes dissection.

A total of 341 participants in three studies (14, 19, 20) reported TME incomplete, and RS significantly reduced the risk of TME incomplete (OR 0.40, 95% CI 0.17, 0.93, *P* = 0.03) (Figure 6A). In addition, circumferential resection margin (CRM) positivity was comparable (OR 0.90, 95% CI 0.63, 1.27, *P* = 0.54) between the RS and LS groups (Figure 6B). Distal margin positive rates were reported in two studies (8, 18), and the pooled results showed no significant difference in the rate of distal margin positive rates between the RS and LS groups (OR 0.60, 95% CI 0.29, 1.22, *P* = 0.16) (Figure 6C). There was no significant difference in the number of nodes dissection between RS and LS (MD 0.69.; 95% CI: −0.43, 1.82, *P* = 0.23) (Figure 6D).

#### 3.3.6 Postoperative status of patients

**Figure 7:**
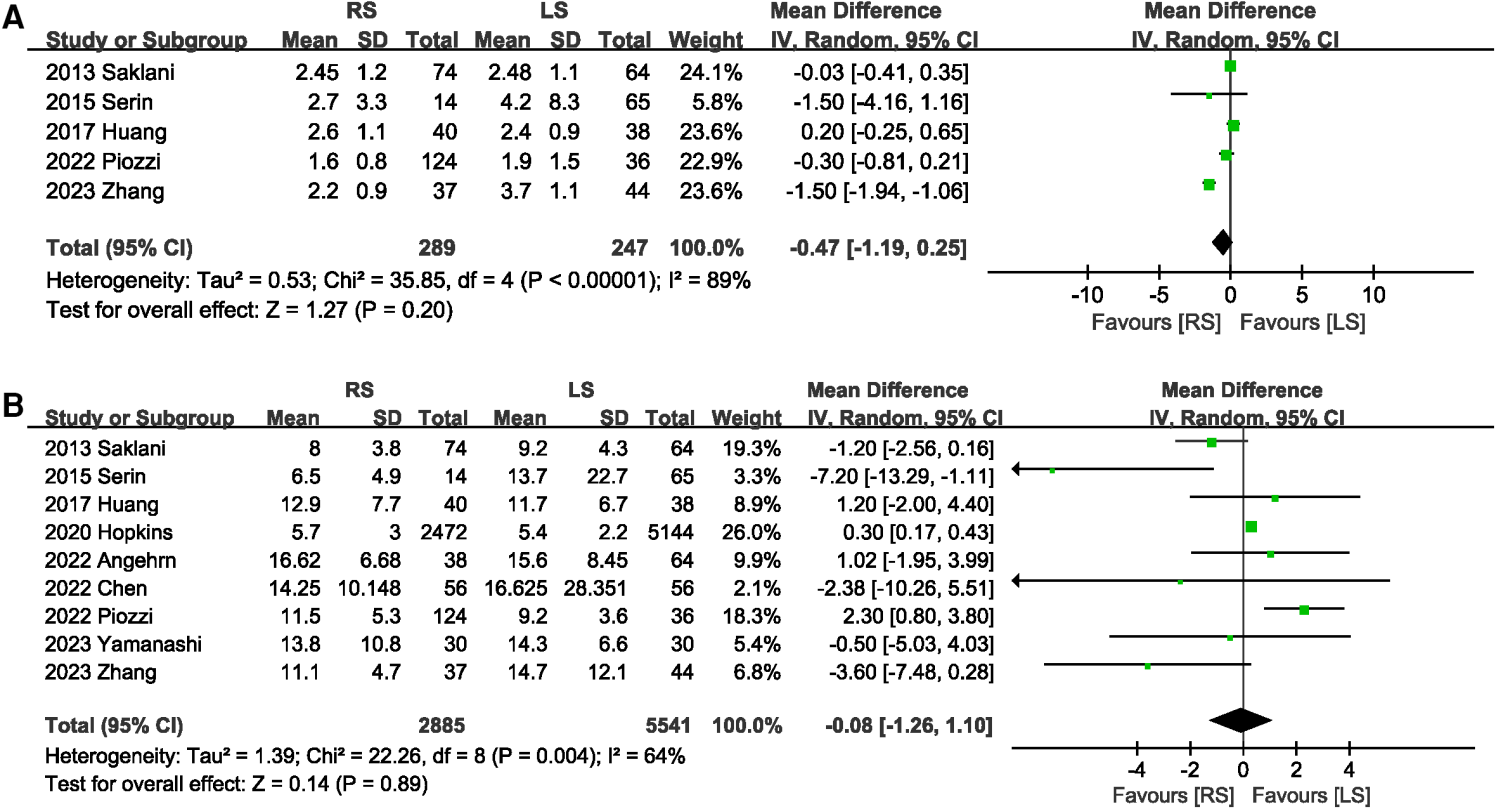
Forest plot of (**A**) the time to first flatus and (**B**) length of hospital stay.

A total of 536 participants in five studies (6, 13–15, 20) reported the time to first flatus. The overall effect indicated that RS did not shorten the time to first flatus (MD −0.47 days; 95% CI: −1.19, 0.25), with significant heterogeneity (*I*^2^ = 89%, *P* < 0.00001) between studies (Figure 7A). Only one study described the first bowel movement time. This study found that RS shortened the time to the first bowel movement. Length of hospital stay was mentioned in nine (6–8, 13–15, 17, 19, 20) studies. No significant difference in the length of hospital stay was observed between the RS and LS groups (MD −0.08 days; 95% CI: −1.26, 1.10) (Figure 7B).

### 3.4 Sensitivity analysis

The results of the sensitivity analysis showed that the total effect size of postoperative complications, CRM positivity, distal margin positive rates, operative time, length of hospital stay, the time of first flatus and defecation, lymph node harvested, and conversion rate were not affected by the elimination of any one study. The total effect size for the blood loss changed when the study by Chen et al. (8) (MD −24.27 ml; 95% CI: −38.42, −10.13, *P* = 0.0008) was excluded.

## 4 Discussion

Our results showed that RS significantly increased the operation time, but it reduced the risk of conversion to open surgery, and improved the completeness of TME compared with LS in patients with rectal cancer after neoadjuvant therapy. In addition, there were no significant differences in postoperative morbidity, mortality, length of hospital stay, blood loss, time to first exhaust, CRM positive rate, lymph node detection rate, and distal margin positive rates between RS and LS.

Due to the lack of obvious clinical symptoms, most patients with rectal cancer are not clearly diagnosed until they have developed to locally advanced stage (21). Patients with advanced rectal cancer often require neoadjuvant therapy. Although neoadjuvant therapy can effectively reduce the tumor stage and local recurrence rate, it is associated with an increase in postoperative complications (21, 22). Previous evidence has shown that the incidence of postoperative complications in patients with rectal cancer treated with neoadjuvant therapy is as high as 21% (23). Similarly, the overall incidence of postoperative complications in our study was 25%. Postoperative complications not only increase the economic burden of patients, but also compromise their oncologic outcomes (21). Theoretically, RS has good maneuvability in the limited pelvic space and provides a good field of view, which may be expected to reduce postoperative morbidity. However, the results of our meta-analysis suggest that robot-assisted surgery does not reduce the risk of postoperative complications and mortality. This is consistent with findings from several recent large RCTs (24–26).

The conversion rate in minimally invasive surgery not only reflects the complexity of surgical techniques, but also has important clinical significance. Previous evidence suggests that conversion to open surgery is associated with an increased risk of postoperative morbidity and local recurrence (7, 27). Conversion rates for laparoscopic rectal cancer surgery range from 9% to 16%, with rates as high as 25% among patients receiving neoadjuvant therapy (7). In a narrow pelvis, the 3D view of the RS system is able to allow the surgeon to clearly identify anatomical structures and gaps to minimize tissue damage caused by the dissection procedure (3). Therefore, RS may be a potential strategy to reduce the rate of conversion to open surgery. The failure of the ROLARR trial to confirm a benefit of RS in reducing conversion to open surgery may be related to the inclusion of patients with higher rectal cancers and the low proportion of patients who received neoadjuvant therapy (25). However, a recently published large RCT showed that RS (10/586) significantly reduced the conversion rate of mid-low rectal cancer surgery compared with LS (23/585) (28). In addition, several recently published meta-analyses have also suggested that RS is associated with lower rates of conversion to open surgery (3, 29). This evidence is consistent with our results. Considering the potential impact of conversion to open surgery on long-term prognosis, future studies are warranted to further explore the impact of RS on the long-term prognosis of rectal cancer patients undergoing neoadjuvant therapy.

A large number of previous studies have shown that RS prolongs the operation time compared with LS (3, 4, 30). Our study also showed that RS was associated with longer operative time, but intraoperative blood loss was comparable between the RS and LS groups. The prolonged operation time may be related to the following two aspects. On the one hand, LS has been widely and skillfully used by surgeons, while RS is relatively a new surgical procedure, and some surgeons may not be as proficient in RS as LS. On the other hand, the setup of surgical instruments for RS takes a lot of time (30). When the time to assemble the machine was excluded, there was no significant difference in the duration of surgery between the two groups (31). With the popularization of RS, the difference in operative time is expected to gradually decrease.

The introduction of TME has improved the oncologic prognosis of patients with rectal cancer. At present, TME has become the gold standard for rectal cancer surgery, and complete TME can reduce the risk of postoperative recurrence (32). Our results suggest that RS improves the TME completeness rate, which may help improve the prognosis of patients with rectal cancer. Similarly, a meta-analysis of 12 studies showed that RS was a better way to achieve complete TME than traditional LS (33). The number of harvested lymph nodes not only determines the accuracy of postoperative staging, but also is related to the prognosis of patients (34). In addition, positive CRM increased the risk of tumor recurrence (35). Our study showed no significant difference between robotic progenitor and laparoscopic groups in the number of lymph nodes harvested and CRM positive rate. These results indicate that RS can achieve pathological results that are noninferior to LS in patients with rectal cancer.

Several studies have suggested that RS may be associated with faster gastrointestinal function recovery and shorter hospital stay (3, 36). The time to first flatus and defecation are the key indicators to evaluate the recovery of intestinal function. Of the 12 studies we included, only 5 studies assessed the time to first flatus and 1 study reported the time to first defecation. The pooled results showed that the time to first flatus was comparable between the RS and LS groups. Due to the limited number of included studies, more studies are needed to evaluate the effects of robots on gastrointestinal function recovery.

Our study has the following strengths. On the one hand, we performed a comprehensive database search without language and time constraints. On the other hand, we included the most recent data, making the pooled results more convincing.

Meanwhile, our meta-analysis has several limitations. First, the studies we included were all retrospective studies, which have the inherent bias of retrospective studies. Second, some measures may have been underpowered to draw firm conclusions. Finally, we included some studies with small samples, which may have potential bias.

In conclusion, based on evidence from NRS, this meta-analysis indicates that RS prolonged the procedure time, but significantly improved the rate of conversion to open surgery and the completeness of TME. In addition, RS and LS were comparable in terms of postoperative morbidity, mortality, length of hospital stay, blood loss, time to first exhaust, CRM positive rate, lymph node detection rate, and distal margin positive rates. In the future, high-quality prospective studies are needed to further explore the impact of RS on rectal cancer patients after neoadjuvant therapy.

## Data Availability

The original contributions presented in the study are included in the article/Supplementary Material, further inquiries can be directed to the corresponding author.
